# Outbreak of Mass Sociogenic Illness in a School Feeding Program in Northwest Bangladesh, 2010

**DOI:** 10.1371/journal.pone.0080420

**Published:** 2013-11-14

**Authors:** Farhana Haque, Subodh Kumar Kundu, Md Saiful Islam, S. M. Murshid Hasan, Asma Khatun, Partha Sarathi Gope, Zahid Hayat Mahmud, A. S. M. Alamgir, M. Sirajul Islam, Mahmudur Rahman, Stephen P. Luby

**Affiliations:** 1 Centre for Communicable Diseases (CCD), icddr,b, Dhaka, Bangladesh; 2 Institute of Epidemiology, Disease Control and Research (IEDCR), Dhaka, Bangladesh; 3 Centre for Food and Waterborne Diseases, icddr,b, Dhaka, Bangladesh; 4 Global Disease Detection Program, Centers for Disease Control and Prevention (CDC), Atlanta, Georgia, United States of America; United (Osaka U, Kanazawa U, Hamamatsu U Sch Med, Chiba U and Fukui U) Graduate School of Child Development, Japan

## Abstract

**Background:**

In 2010, an acute illness outbreak was reported in school students eating high-energy biscuits supplied by the school feeding programme in northwest Bangladesh. We investigated this outbreak to describe the illness in terms of person, place and time, develop the timeline of events, and determine the cause and community perceptions regarding the outbreak.

**Methods:**

We defined case-patients as students from affected schools reporting any two symptoms including abdominal pain, heartburn, bitter taste, and headache after eating biscuits on the day of illness. We conducted in-depth interviews and group discussions with students, teachers, parents and community members to explore symptoms, exposures, and community perceptions. We conducted a questionnaire survey among case-patients to determine the symptoms and ascertain food items eaten 12 hours before illness onset, and microbiological and environmental investigations.

**Results:**

Among 142 students seeking hospital care, 44 students from four schools qualified as case-patients. Of these, we surveyed 30 who had a mean age of 9 years; 70% (21/30) were females. Predominant symptoms included abdominal pain (93%), heartburn (90%), and bitter taste (57%). All students recovered within a few hours. No pathogenic *Vibrio cholerae*, *Shigella* or *Salmonella* *spp*. were isolated from collected stool samples. We found no rancid biscuits in schools and storage sites. The female index case perceived the unusually darker packet label as a “devil’s deed” that made the biscuits poisonous. Many students, parents and community members reported concerns about rumors of students dying from biscuit poisoning.

**Conclusions:**

Rapid onset, followed by rapid recovery of symptoms; female preponderance; inconsistent physical, microbiological and environmental findings suggested mass sociogenic illness rather than a foodborne or toxic cause. Rumours of student deaths heightening community anxiety apparently propagated this outbreak. Sharing investigation results and reassuring students and parents through health communication campaigns could limit similar future outbreaks and help retain beneficiaries’ trust on nutrition supplementation initiatives.

## Introduction

Outbreaks of mass sociogenic illness (MSI) also known as epidemic hysteria or mass psychogenic illness (MPI) have been reported in schools worldwide [1-3]. Rapid onset and quick spread of symptoms among members of a cohesive group without any organic basis tends to signal mass sociogenic illness. These are frequently characterized by a rapid recovery, a higher tendency of affecting females, and transmission through visual or oral communication channels [4-6].

School meal programs are common public health interventions implemented in both low- and high-income settings to improve educational quality and efficiency by alleviating short-term hunger and fulfilling energy and micronutrient deficiencies of students [7-12]. The World Food Programme (WFP) supports school meal programs in more than 60 countries worldwide [13]. In Bangladesh, the WFP provides a daily snack of eight fortified high-energy biscuits (HEBs) in a single packet to primary school students in food-insecure areas [14].

Insufficient sanitation and hygiene, faulty storage in hot and moist environments, and inadequate food safety monitoring practices had rarely caused rancidity and contamination of food supplements leading to gastrointestinal illnesses in the past [15]. In July 2013, at least 25 children died after consuming contaminated school meals in the state of Bihar, India [16]. On the other hand, mass sociogenic illness outbreaks in schools have been frequently reported worldwide [1,3,17-21]. Several mass sociogenic illness outbreaks associated with HEBs were reported in the mass media in Bangladesh during 2009 [22]. While the beneficial impacts of school meals have been widely evaluated, there is limited published evidence on mass sociogenic illness outbreaks implicating school meals [14,23-28]. Mass sociogenic illness outbreaks potentially related to health interventions could generate widespread anxiety, reduce acceptability of programs and interrupt public health efforts among affected communities, and also negatively impact beneficiaries worldwide [19,29].

During October 2010, newspapers reported an outbreak of acute symptoms after eating HEBs among students from five primary schools of two sub-districts of Gaibandha District in northwest Bangladesh. The local health authority investigated and reported the occurrence to the Institute of Epidemiology, Disease Control and Research (IEDCR) of the Ministry of Health as mass psychogenic illness. Since several similar outbreaks implicating these biscuits were reported, the WFP requested an independent investigation to confirm the cause. A collaborative team composed of epidemiologists, anthropologists, physicians, environmental microbiologists, laboratory experts and field workers from IEDCR and the International Centre for Diarrhoeal Disease Research, Bangladesh (icddr,b) conducted an investigation to describe the illness in terms of person, place and time, determine the cause, document the timeline of events, and describe the community perceptions.

## Methods

### Ethics statement

We advised school teachers to reassure the children and their families that long-term sequelae from the current illness episodes were unlikely. We sought permission from the headmasters of respective schools to interview students and teachers. Field investigators sought verbal informed consent from the adult participants and verbal assent from child respondents. As this investigation was a part of an emergency response to an immediate public health threat and the primary purpose of this activity was to identify, characterize, and control the illness outbreak, this investigation was exempted from review by an independent human subjects committee. However, this investigation was approved by and conducted in collaboration with the Government of the People's Republic of Bangladesh.

### Epidemiological investigation

**Figure 1:**
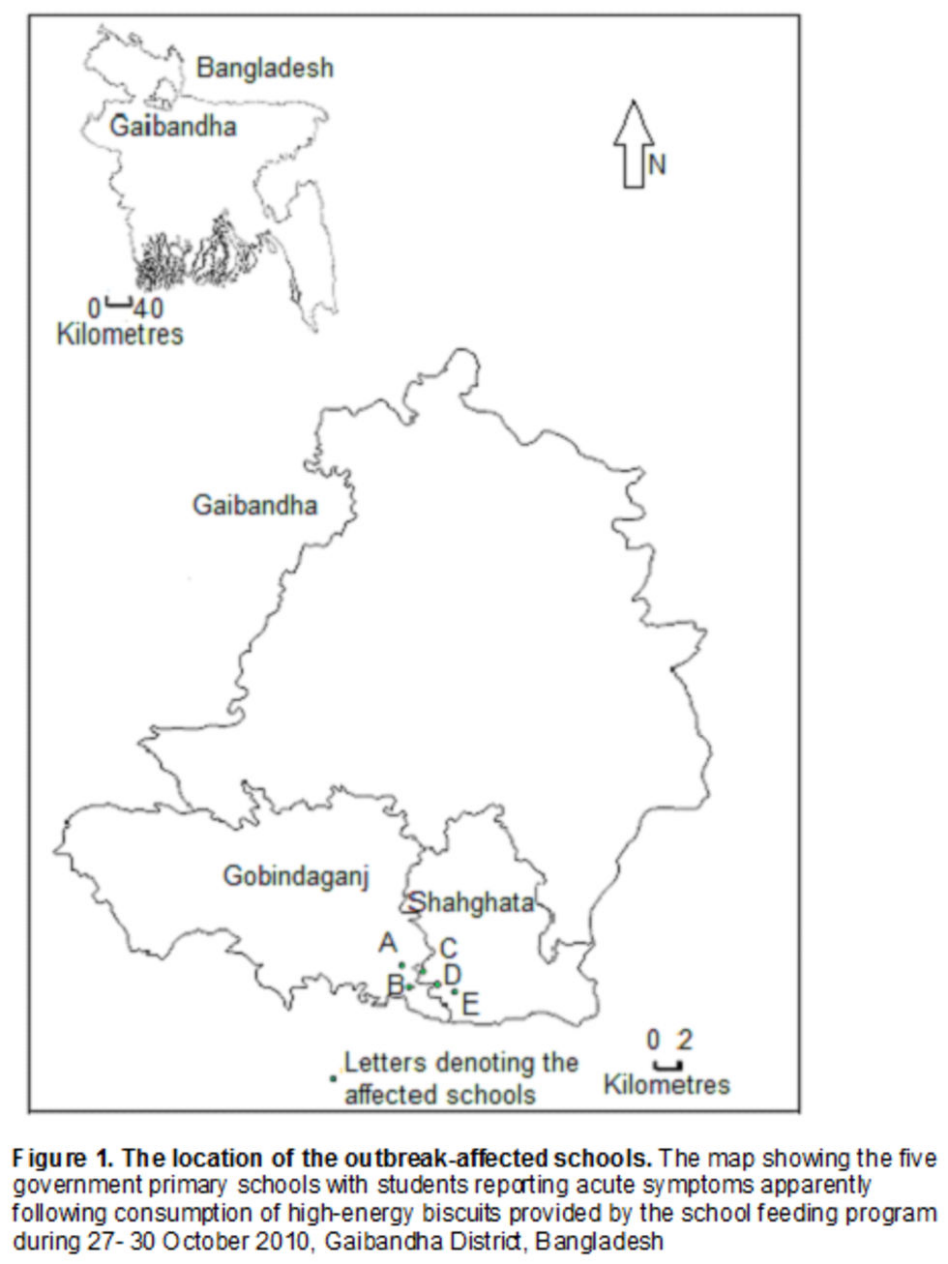
The location of the outbreak-affected schools. The map showing the five government primary schools with students reporting acute symptoms apparently following consumption of high-energy biscuits provided by the school feeding program during 27−30 October 2010, Gaibandha District, Bangladesh.

The team visited the affected area during 6−8 November 2010 and conducted unstructured interviews with the local health authority to verify the outbreak and to generate hypothesis about the potential cause. The team visited the five affected schools (Figure 1) and conducted unstructured interviews with affected students and teachers to construct a timeline of events. 

The team defined a case-patient as any student from the affected schools who reported any two symptoms including nausea, vomiting, diarrhoea, heart burn, bitter taste, headache, neck pain, or chest tightness following consumption of HEBs on the day of the illness. To ascertain the food items that were eaten during the 12 hours prior to illness onset and identify symptoms, the team surveyed the case-patients in four of the five affected schools using pretested, structured questionnaires developed by the authors. Field anthropologists visited three affected schools and conducted one group discussion with seven female and three male case-patients from the index school, and 14 open ended interviews with at least three students from each school to explore exposure history, symptoms, care-seeking practices and perceptions about the outbreak. They conducted 7 in-depth interviews with at least one teacher from each school and cross-checked the exposure history, symptoms and signs, and timeline of events. They visited 8 case-households and interviewed five mothers, two fathers and one grandmother to explore case-patients’ past illness history and determine family members’ perceptions about the outbreak.

### Environmental investigation

To understand the storage, handling and distribution process of HEBs, the team inspected the warehouse where the biscuits were stored before distribution to different schools in the district. The team then inspected the *storage school*, where the biscuits were stored temporarily for about a month following delivery from the warehouse and before being supplied to individual primary schools in the sub-district. Environmental microbiologists on the team explored local water supply and sanitation infrastructure and collected water samples from the affected schools’ tube wells. Given that contaminated water was regarded as an important vehicle for diarrhoea transmission in Bangladesh [30,31], team members collected water from tube wells and drinking water storage containers of affected students’ households. However, water contamination was very common in Bangladesh and apparently any water was likely to exceed World Health Organization's recommended microbial levels. Thus to conduct a comparative analysis of microbial quality of drinking water, we collected water from households of age- and sex-matched controls. We recruited controls from the students belonging to the same classes and schools as case-patients who had eaten biscuits on the day of the outbreak but did not report having any symptoms.

### Laboratory investigation

All collected water samples were stored in cool boxes with temperatures maintained at 4−8 °C. The samples were transported to icddr,b’s Environmental Microbiology Laboratory and analyzed within 24 hours of collection. The samples were tested for indicator bacteria such as faecal coliforms, faecal streptococci, *Vibrio cholerae* and *Clostridium perfringens*. 

Field investigators collected biscuit packets from the same delivery lot as the ones that had allegedly caused the outbreak from four affected schools, the warehouse and the storage school to test for microbial contamination and to determine the protein, fat, fibre, calcium, iron and moisture contents. Laboratory technicians collected stool samples from case-patients and their age- and sex-matched controls to test for common enteric pathogens. 

## Results

### Timeline of events

Each affected government primary schools, which, we refer to as: A, B, C, D, and E, had 200-300 students studying in classes I to V. HEBs were distributed at around 10:00 AM in morning shifts and 12:30 PM in day shifts.

#### 27 October 2010 (10:30 AM): Events in School A in Gobindaganj

The teachers distributed the biscuits to 256 students. The female index case from class III perceived the variation in the colour from blue to black in the biscuit packet’s label supplied to her on that day. The index case, whom the community reportedly considered as a child under an evil spell (*Jiner asor*), reported that the colour change was an evil deed. She also shared her concern with two classmates that the black colour signaled to her that those biscuits were poisonous. She requested the student representative, who was distributing biscuits that day on behalf of the teacher, to exchange her packet for a different one. She directly requested the teacher but the teacher refused and insisted that she eat the biscuits that were already supplied to her. At 11:00 AM, within 30 minutes after eating the biscuits, the index case complained abdominal pain, blurred vision, headache, and fainted. Within a few minutes, 18 students from the same class developed similar symptoms. Nine students from class I and 11 students from class II became sick in another 10 to 15 minutes. Parents of many asymptomatic students hurriedly took their children to the hospital out of fear that they might also fall sick. Students from classes IV and V (in the day shift) were not given any HEBs and none reported any symptoms. 

#### Response to the incident on 27 October, 2010

Following the incident, the WFP in consultation with the local health authority suspended the biscuit distribution in all beneficiary schools within the affected sub-district until the cause of the outbreak was confirmed.

#### 28 October 2010 (11:00 AM): Events in School B in Gobindaganj

The teachers reported that they did not distribute any biscuit on that day. At around 11:00 AM, 3 students of class III developed similar symptoms despite not eating HEBs on that day. According to the class teacher, a group of agitated community residents including local leaders rushed to the affected classroom and criticized the supplier factory for providing poisonous biscuits. They also blamed the school and the WFP authorities for not taking the initiative to change the biscuit factory. At around 11:15 AM, another 30 students complained similar symptoms. The affected students sought care from the primary healthcare hospital and were discharged within a few hours following complete recovery. 

#### 30 October 2010 (11:00 AM): Events in School C in Shahghata

On the evening of 29 October 2010, the first affected female case of school C heard the news of students dying from biscuit poisoning in school A from her aunt by mobile phone. At around 9:00 AM the next morning, she shared this story with her classmates. Later, at approximately 11:00 AM, students from school C started experiencing similar symptoms within 30 minutes of biscuit distribution. The news had spread to nearby schools and community residents and the media rushed to the school. The teachers reported that when children saw their mothers crying, more students started experiencing similar symptoms.

#### 30 October 2010 (1:00 PM): Events in School D in Shahghata

Seven students of classes III─IV from the day shift developed symptoms after eating the biscuits at 12:30 PM. None of the students from the morning shift (classes I and II) were affected. 

#### 30 October 2010 (2:00 PM): Events in School E in Shahghata

Three day shift students became sick after eating the biscuits. 

#### Response to the incident on 30 October, 2010

The WFP and local health authorities decided to suspend the biscuit distribution initiative in all beneficiary schools located within any sub-district of Gaibandha district until confirmation of cause. 

### Descriptive epidemiology

142 students from 5 schools reported symptoms and sought hospital care. Of these, we identified 44 case-patients from four schools. The students from school B had not eaten biscuits on the day of the illness and so none met our case definition and none were included in the survey (Table 1). 

**Table 1:** The distribution of affected students within the five government primary schools in Gaibandha District, Bangladesh.

School designation, sub-district	Proportion of students reported suffering from the outbreak among students eating the biscuits, (%)	Number of affected students meeting case definition N=44, (%)	Proportion of case-patients among students eating the biscuits, (%)
School A, Gobindaganj	39/256 (15)	19 (43)	19/256 (7)
School B, Gobindaganj	33/253 (13)	0 (0)	0/253 (0)
School C, Shahghata	19/161 (12)	7 (16)	7/161 (4)
School D, Shahghata	30/252 (12)	15 (34)	15/252 (6)
School E, Shahghata	21/251 (8)	3 (7)	3/ 251 (1)
Total	142/1173 (12)	44 (100)	44/1173 (4)

The affected students had visited primary care hospitals with reported acute physical symptoms apparently after consumption of high-energy biscuits provided by the school feeding program during 27−30 October 2010

We also did not survey 98 students who sought hospital care but had reported little or no physical symptoms. We administered the face-to-face survey to 30/44 case-patients who were present in their respective schools during the field investigation. Their mean age was 9 (range: 7−12) years and 70% (21/30) were females. Predominant symptoms included abdominal pain, burning sensation and bitter taste in the throat (Table 2). 

**Table 2:** Characteristics and symptoms reported by case-patients experiencing acute symptoms following consumption of high-energy biscuits supplied to students by the school feeding program during 27−30 October 2010, Gaibandha District, Bangladesh.

Characteristics of case-patients^a^		Number of persons, (%) N=30^b^
Sex	Male	9 (30)
	Female	21 (70)
Education	Class 1	7 (23)
	Class 2	11(38)
	Class 3	4 (13)
	Class 4	7 (23)
	Class 5	1 (3)
Symptoms	Abdominal pain	28 (93)
	Burning sensation in the throat	27 (90)
	Bitter taste in the throat	17 (57)
	Headache	13 (43)
	Nausea	12 (40)
	Generalized weakness	9 (30)
	Vomiting	6 (20)
	Diarrhoea (defined as three of more loose stools per day)	6 (20)
	Chest tightness	5 (17)
	Neck pain	3 (10)
	Vertigo	2 (7)
	Loss of consciousness	2 (7)
	Others (blurring of vision, throat ache, dry mouth, chest pain)	7 (23)
Feeding history	Had breakfast in the morning before coming to school	29 (97)
	Had eaten 5 - 8 biscuits at the same time	22 (73)

a Case-patients defined as any student from the affected schools who reported any two symptoms including nausea, vomiting, diarrhoea, heart burn, bitter taste, headache, neck pain, or chest tightness following consumption of high-energy biscuits on the day of the illness.

b Out of 44 case-patients, we surveyed the 30 students who were present in the schools during the investigation.

The mean number of biscuits consumed was 6 (range: 2−8). The median interval between biscuit consumption and symptom onset was 30 minutes. Twenty (6/30) percent complained that biscuits had either tasted bitter or smelt bad or looked darker than usual (dark brown/black) on that day. 90% (17/19) of the affected students from School A including the index case reported that they had noticed an unusual black ink labeling on the biscuit packets supplied to them on that day. Of the 109 students from the four schools who had sought hospital care, 107 were discharged within a few hours. Two students who had reported fever and lived far away from the hospital were discharged the day following admission.

### Perceptions about the illness outbreak

The students, parents, teachers and the community residents of schools A, C, D, and E reported many causes including the black ink on packet labels, altered biscuit colour, bitter or medicinal taste, moldy smell and faulty storage were responsible for the outbreak. One headmaster reported that junior teachers had sometimes complained that the biscuits became moist after 20 to 25 days of storage during rainy seasons. The teachers reported that parents expressed fear and lack of trust about public health programs in general including the school meal program. However, one out of the five mothers interviewed did not believe that the biscuits had caused the outbreak. She expressed, 

“*My children are taking the biscuits since 2007 and they never became sick*.

The headmasters and the teachers explained that the rumor of students dying after eating biscuits at the index school had spread rapidly among the villagers since all the schools were located in nearby villages. The physicians who had attended the affected students reported the event as a mass psychogenic illness outbreak. Informal discussions with five management committee members of school B, revealed that a group of local leaders, who the respondents perceived as recipients of financial benefits from competitor factories, had previously expressed dissatisfaction with the current producer factory and had recommended to the local WFP authority and school management committee to change the existing manufacturer and include their preferred factories into the program. According to the management committee, this group had been spreading rumours of student deaths following consumption of unhygienic biscuits within the community to defame the existing manufacturer factory. The interviewed local healthcare providers believed that the rumours probably added to the students' anxiety leading to the outbreak among students even without eating biscuits.

### Community’s response to the outbreak at the index school

Immediately after students began having symptoms, the teachers advised all students to stop eating and to throw the remaining biscuits into the adjacent ditch. Community residents and parents who were near the school during the episodes rushed to the school. Parents from villages also rushed to the school. A few guardians fed the sick students boiled olives, tamarinds and pickles, which they believed were useful remedies for any poisons that were likely to be present in the biscuits. The teachers hired rickshaw vans to take the sick students to the nearest government sub-district hospital. During transport, many parents poured water on the heads of the sick students. Some parents were crying loudly. Some community residents thought that the students who had fainted were already dead. Many community residents reported that they had heard the rumour of student deaths in the index school after eating the biscuits, which increased their fear. They reported sharing their concerns with many relatives, neighbours and also with people from nearby villages. 

### Environmental findings

#### Manufacture, storage, maintenance and distribution of the high-energy biscuits

The biscuits were manufactured in approved local industries that met WFP quality specifications. WFP food inspectors were involved in the quality control. The teachers and local WFP employees calculated monthly requirements of individual biscuit packets for each school and sent out a requisition to the WFP regional office. The biscuits, packaged in sealed cartons containing 100 individual packets, were stored in a standard warehouse in the district following delivery from the regional WFP office every month. The biscuits were often stored for a day or two in the storage school before being delivered to individual primary schools. The school authority then stored the cartons in the classrooms that reportedly had good ventilation for daily distribution among the students. The school management committee including teachers, parent representatives, community leaders, administrative officials, and non-governmental organization local service providers were responsible for overseeing the distribution, hygiene, sanitation and storage process in each primary school. We found that the warehouse was well-maintained. The classrooms of both storage schools had adequate ventilation. The field team found no rancid biscuits in the district warehouse or other school storage sites. We learnt from local WFP officials that one factory had changed the labels’ colour to black on some biscuit packets when they ran out of blue ink. The factory authority considered this a minor change, as it was neither related to biscuit or packaging quality, and consequently did not inform WFP immediately. 

#### Water samples test results

Several water samples from schools and households of case-patients and controls did not meet the World Health Organization’s guidelines for microbial quality of drinking water (Table 3). 

**Table 3:** Bacteriological test results of water samples collected from various sources during the acute illness outbreak affecting primary school students after consumption of high-energy biscuits during 27-30 October 2010, Gaibandha District, Bangladesh.

Source of sample	Number of samples exceeding the limit of faecal coliforms^a^	Number of samples exceeding the limit of faecal streptococci^b^	Number of samples exceeding the limit of *Vibrio cholerae* or *Clostridium perfringes* ^c^
School tube well [N=4]	1	2	0
Stored drinking water (case-patients^d^) [N=26]	18	21	2
Tube-well (case-patients) [N=25]	4	9	1
Stored drinking water (controls^e^) [N=29]	18	24	5
Tube-well (controls) [N=28]	5	7	1

a Maximum allowable limit of faecal coliform-0 colony forming unit (cfu)/100ml─WHO guideline for drinking water

b Maximum allowable limits of faecal streptococci-0 cfu/100ml─WHO guideline for drinking water

c Maximum allowable limits of *Vibrio cholerae* and *Clostridium perfringes*−0 cfu/100 ml─WHO guideline for drinking water

d Students who experienced at least two gastrointestinal symptoms after consuming high energy biscuits on the day of the illness outbreak

e Students who ate the high energy biscuits on the day of the illness outbreak but did not report symptoms

### Laboratory findings

No pathogenic *Vibrio cholerae*, *Shigella* or *Salmonella* were isolated from stool samples of affected students (N=26) and controls (N=28). Parasites including *Giardia lamblia*, *Entamoeba histolytica/Entamoeba dispar*, *Ascaris lumbricoides* and *Enterobius vermicularis* were found in stool samples from four case-patients and five controls. Product composition and microbiological analyses of biscuits revealed no abnormalities (Table 4). 

**Table 4:** Microbial and product composition analyses report of high-energy biscuits.

Ingredients	Test results	Acceptable levels as per the WFP’s technical specifications of high-energy biscuits
Moisture content, %	4.00	4.50 (Max)
Protein, g/100 g dry matter	11.70	15.00 (Max)
Fat, g/100 g dry matter	14.20	15.00 (Min)
Acid insoluble ash, %	0.04	0.08 (Max)
Crude fibre, g/100 g dry matter	1.30	2.30 (Max)
Calcium, mg/100 g	191	250.00 (Min)
Iron, mg/100 g	15.80	18.00 (Min)
Aerobic bacteria, cfu/g	10-1600	10, 000 (Max)
Total coliforms, cfu/g	0	10 (Max)
Faecal coliforms, cfu/g	0	0 (Max)
Faecal streptococci, cfu/g	0	0 (Max)

• Product analysis done at the Bangladesh Standards Testing Institute (BSTI)

• Microbial analysis done at the Environmental Microbiology Laboratory of icddr,b

• cfu/g stands for colony forming unit per gram change

These biscuits were collected from the storage sites and from a student of one of the four primary schools with students reporting acute symptoms following consumption of high-energy biscuits during 27−30 October 2010 and from the World Food Programme's (WFP) district warehouse (N=12), Gaibandha District, Bangladesh

## Discussion

Rapid onset of symptoms followed by rapid recovery, female preponderance together with physical, laboratory and environmental findings inconsistent with a diagnosis of microbial contamination or toxicity suggested that mass sociogenic illness rather than a food-borne illness following school meals was the primary cause of this outbreak [1,3,5,18,32]. The darker labeling on the packet perceived by the index case as a demonic activity that signaled to her that those biscuits were poisonous, altered biscuit colour, and the sight of the index case falling sick apparently triggered this outbreak at the index school. The spread of illness story at the index school through a phone call to a student of another school, and the rapid spread of rumor of students dying after eating biscuits to nearby primary schools in northwest Bangladesh contributed to the collective anxiety that apparently propagated this outbreak.

Public health program beneficiaries living in adverse conditions caused by food insecurity, disasters or complex humanitarian emergencies are often stressed [33]. As stress renders individuals susceptible to psychogenic illnesses [6,34,35], mass sociogenic illness outbreaks are likely to affect health program beneficiaries. The reporting of several mass sociogenic illness outbreaks implicating HEBs during the last two years in Bangladesh also indicated that mass sociogenic illness outbreaks could recur in these vulnerable settings.

Acute illness episodes within schools, having minimal physical findings with no readily identifiable environmental cause, signaled sociogenic illness [18]. However, several food-borne outbreaks initially considered as mass sociogenic illness were later attributed to toxic poisoning [20,21,36]. Alleviating anxiety associated with psychogenic illness thus warrants prompt and reliable investigation before attributing unusual illness episodes in school children to epidemic hysteria. However, establishing the diagnosis through exclusion of multiple potential causes is difficult, lengthy, often contentious [6,18,34,37], and frequently requires considerable expertise of disciplines beyond the health sector. 

We conducted this investigation one week after the outbreak onset, which could have introduced recall bias that particularly affected number of biscuits consumed and nature of symptoms. However, the probability of recall bias was relatively low as we verified information by cross-checking with different sources using data collection methods in both qualitative and quantitative domains and because the incident was unusual. Previous studies have shown that rapid and incomplete investigations followed by dismissive approaches may be harmful to effective management of mass sociogenic illness outbreaks linked to public health interventions [19,29,38]. However, prolonged exhaustive investigations in past mass sociogenic illness outbreaks had been reported to render affected communities suspicious about investigators' objectives; raised concern that a serious cause was being covered up; stigmatized affected people and resulted in relapse [32,34,39,40]. As a result, previous authors have suggested that exhaustive probing into the cause were unnecessary once professionals in authority have recognized mass sociogenic illness as the cause [19,29,41,42]. Given the complex interplay of stakeholders with different social, financial and political agenda in school B, we did not include these students in our survey. Given the inherent multi-factorial challenges surrounding mass sociogenic illness outbreaks, we chose not to conduct a case-control study in this outbreak investigation, though enrolling all students from the five affected schools in an extensive investigation would have strengthened the evidence to establish the diagnosis of mass sociogenic illness. Instead, we conducted a descriptive study, where we adopted a mixed methods approach and triangulated the findings of quantitative survey with the qualitative, laboratory and environmental investigations. Mixed methods combining quantitative and qualitative research paradigms are now increasingly used to answer complex research questions to generate new insights in a constructivist approach [43,44]. 

We found that more than half of the water samples collected from both case-patients and controls failed to meet the water quality requirements of the World Health Organization. However, we did not find any common gastrointestinal pathogens in stool samples of either case-patients or controls. In addition, none of the affected students had any symptoms suggestive of gastro-intestinal infections. In this way, we concluded that it was unlikely that microbial contamination of drinking water contributed to this outbreak. Although we did not find any evidence that collected biscuits were rancid or contaminated, the possibility of some degree of microbial or chemical contamination cannot be completely ruled out, as we did not collect the biscuits on the outbreak days. The biscuits were already removed and discarded by the time we reached the affected schools. Moreover, certain food additives if ingested in large amounts with food can cause symptoms, including headache and chest discomfort, of varying severity [45]. Due to limited laboratory capacity in Bangladesh, we could not test biscuits for food additives. Though Atomic Energy Commission has some capacity for toxicological analysis of food, the lack of a mechanism to rapidly engage disciplines outside of the health sector for a multidisciplinary food safety emergency response prevented timely utilization of the expertise. 

The widespread fear and anxiety associated with mass sociogenic illness outbreaks can hamper nutrition supplementation initiatives, including feeding programs, which aid in combating the continuing high burden of malnutrition in food-insecure countries such as Bangladesh [23,46-48]. Moreover, epidemic hysteria associated with supplementary meals are likely to recur in communities facing the rising threat of food insecurity, hunger and malnutrition in the face of a changing global climate [49]. Under the circumstances, rapid multidisciplinary investigation of outbreaks implicating school meals could help establish the psychogenic cause. Widespread dissemination of investigation reports could reassure affected school communities including students, limit the impact of additional outbreaks, and help retain beneficiaries’ trust in nutrition supplementation efforts worldwide. 

### Ethical approval

We advised school teachers to reassure the children and their families that long-term sequelae from the current illness episodes were unlikely. We sought permission from the headmasters of respective schools to interview students and teachers. Field investigators sought verbal informed consent from the adult participants and verbal assent from child respondents. As this investigation was a part of an emergency response to an immediate public health threat and the primary purpose of this activity was to identify, characterize, and control the illness outbreak, this investigation was exempted from review by an independent human subjects committee. However, this investigation was approved by and conducted in collaboration with the Government of the People's Republic of Bangladesh.
